# Supplementary material for: Supervised Learning for Predictive Pore Size Classification of Regenerated Cellulose Membranes Based on Atomic Force Microscopy Measurements
Source: Materials (Basel). 2021 Nov 8;14(21):6724. doi: 10.3390/ma14216724 (PMC8588053; doi:10.3390/ma14216724)
Supplement: Supplementary file 1 [file materials-14-06724-s001.zip › materials-1397062-supplementary.pdf]

Supplementary Materials

Supervised Learning for Predictive Pore Size Classification of Regenerated Cellulose Membranes Based on Atomic Force Microscopy Measurements

Alex Hadsell [1], Huong Chau [1,2], Richard Barber, Jr. [2,3], Unyoung Kim [1,2] and Maryam Mobed-Miremadi [1,*]

1 Department of Bioengineering, Santa Clara University, Santa Clara, CA 95053, USA; ahadsell@scu.edu (A.H.); hchau@alumni.scu.edu (H.C.); ukim@scu.edu (U.K.)
2 Center for Nanostructures, Santa Clara University, Santa Clara, CA 95053, USA; rbarber@scu.edu
3 Department of Physics, Santa Clara University, Santa Clara, CA 95053, USA
* Correspondence: mmobedmiremadi@scu.edu; Tel.: +1-408-554-2731

Citation: Hadsell, A.; Chau, H., Barber, R., Jr..; Kim, U.; Mobed-Miremadi, M. Supervised Learning for Predictive Pore Size Classification of Regenerated Cellulose Membranes Based on Atomic Force Microscopy Measurements. *Materials* **2021**, *14*, 6724. https://doi.org/10.3390/ma14216724

Academic Editor(s): Claudio Canale; Christian Müller

Received: 11 September 2021
Accepted: 4 November 2021
Published: 8 November 2021

Publisher's Note: MDPI stays neutral with regard to jurisdictional claims in published maps and institutional affiliations.

Copyright: © 2021 by the authors. Licensee MDPI, Basel, Switzerland. This article is an open access article distributed under the terms and conditions of the Creative Commons Attribution (CC BY) license (http://creativecommons.org/licenses/by/4.0/).

Shown below is an accuracy error of 1.5 nm associated with the calibration block. The manufacturer rating of the block is set 20 ± 1.5 nm.

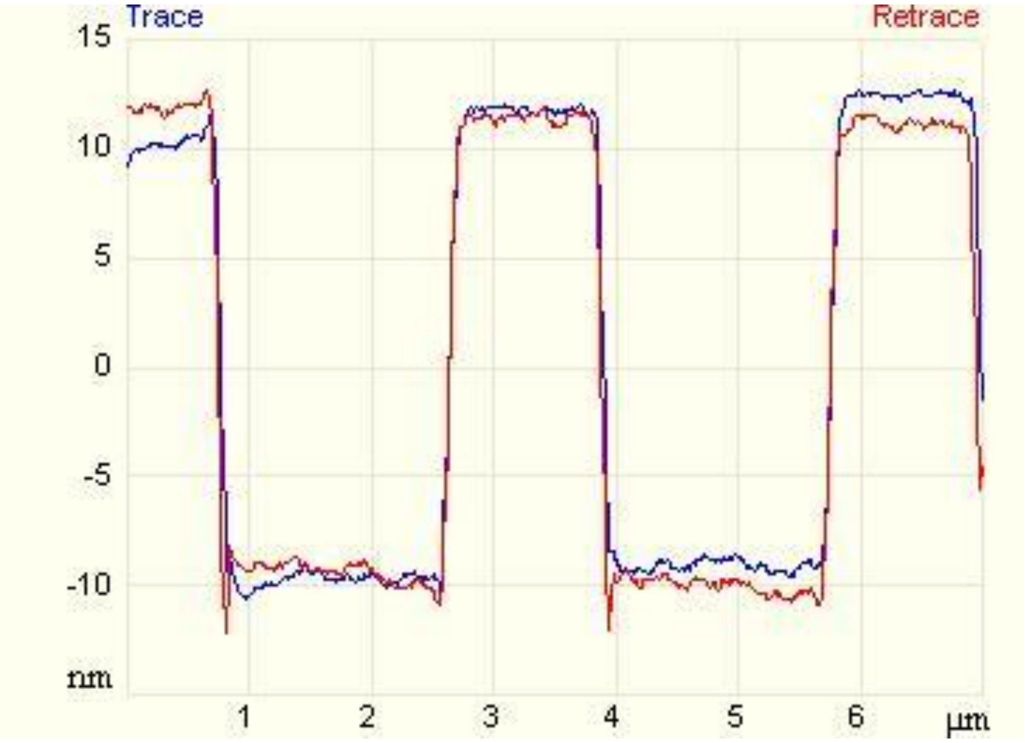

Figure S1. AFM scan corresponding to the calibration block. The step height for the calibration standard was measured to be 21.4 ± 1.5 nm and a period (pitch) of 3 µm ± 0.01 µm.

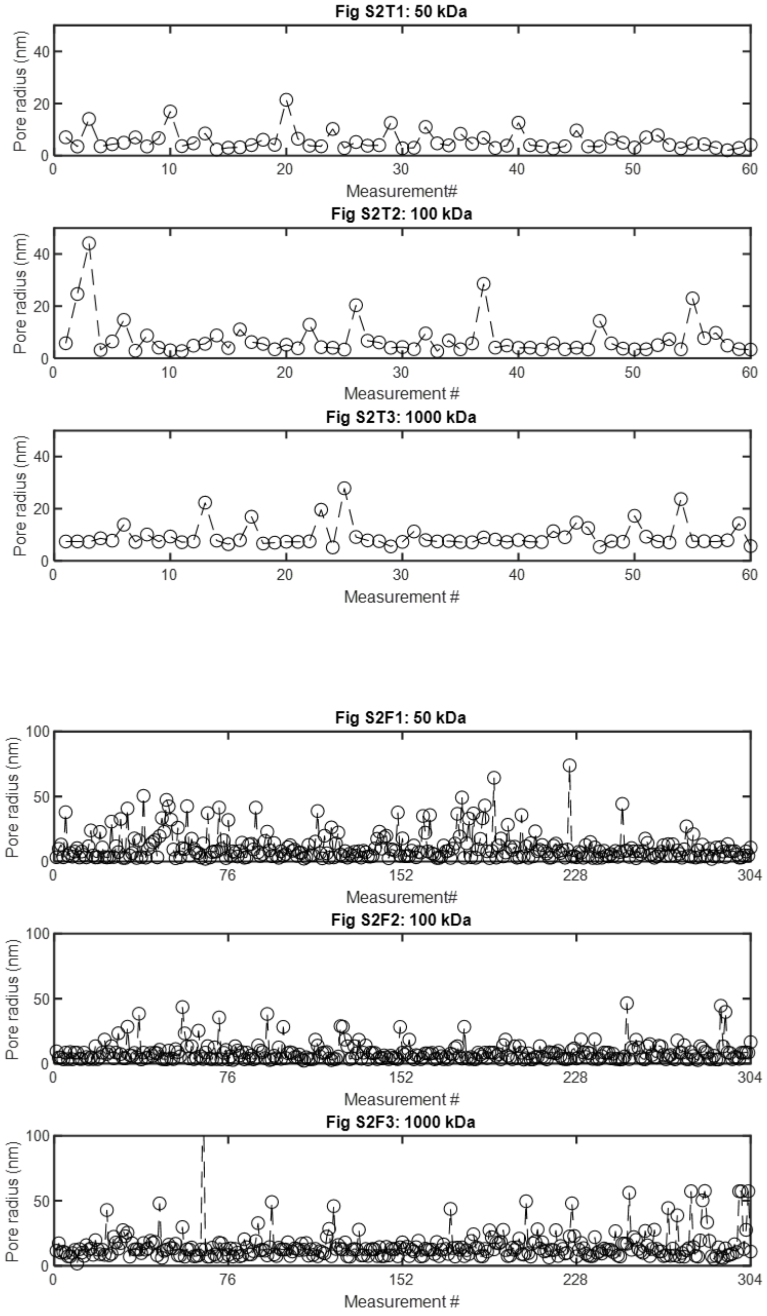

Figure S2. **(A)** Raw data for AFM pore size captured (T1–T3 tapping mode; F1–F3 fluid mode) chronologically across the examined sections (5 sections for tapping mode, 4 sections for fluid mode) showing random spikes across all 3 examined RC membranes. This data was subjected to the Fourier analysis for the detection of periodic instrument and environmental drifts ruled out due to poor adjusted correlation coefficients (R^2_{adj}<<1). Results are summarized in Table 3.

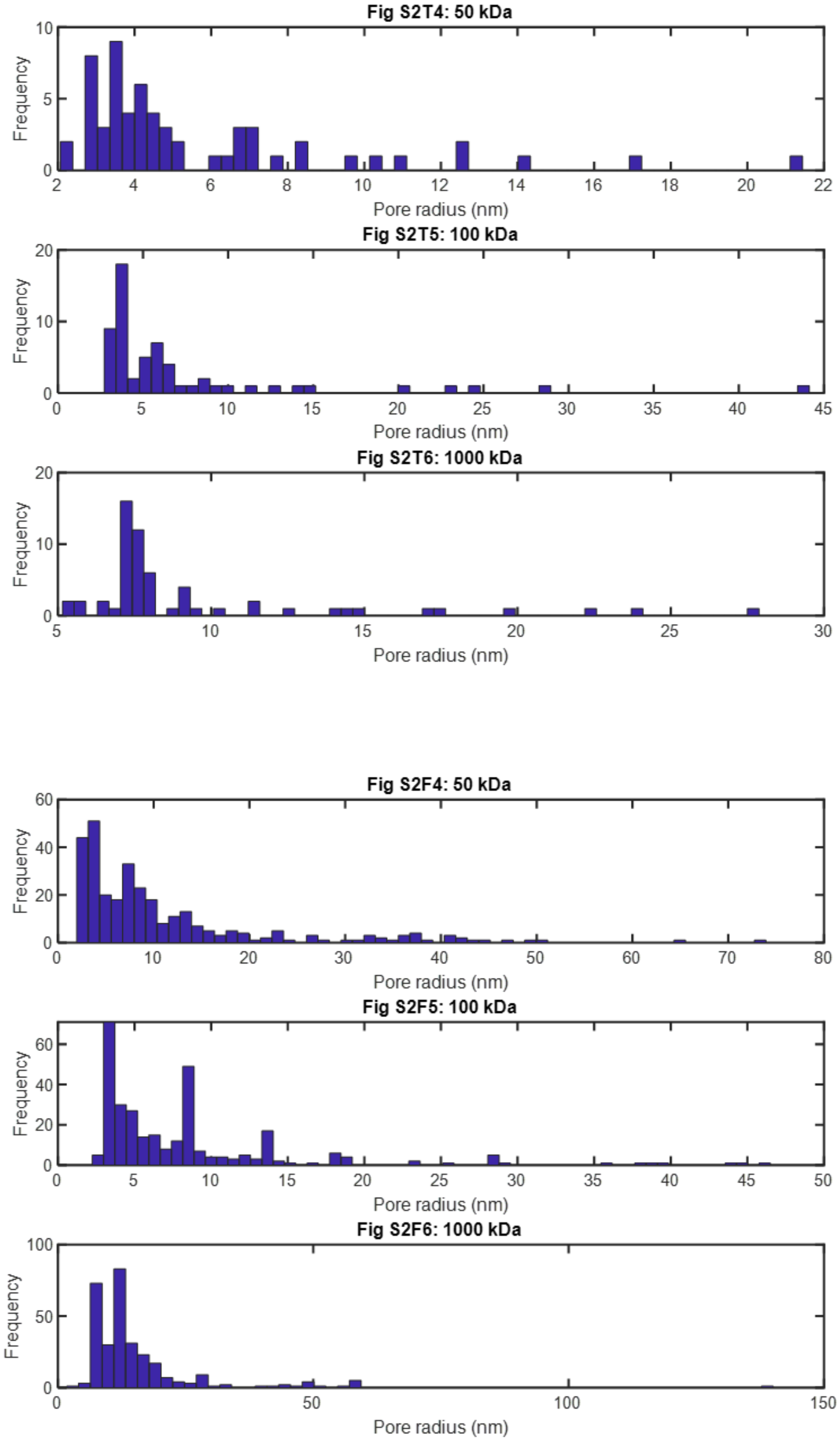

Figure S2. (B) Pore size frequency distribution (T4–T6 tapping mode; F4–F6 fluid mode) indicative of lack of Normality.

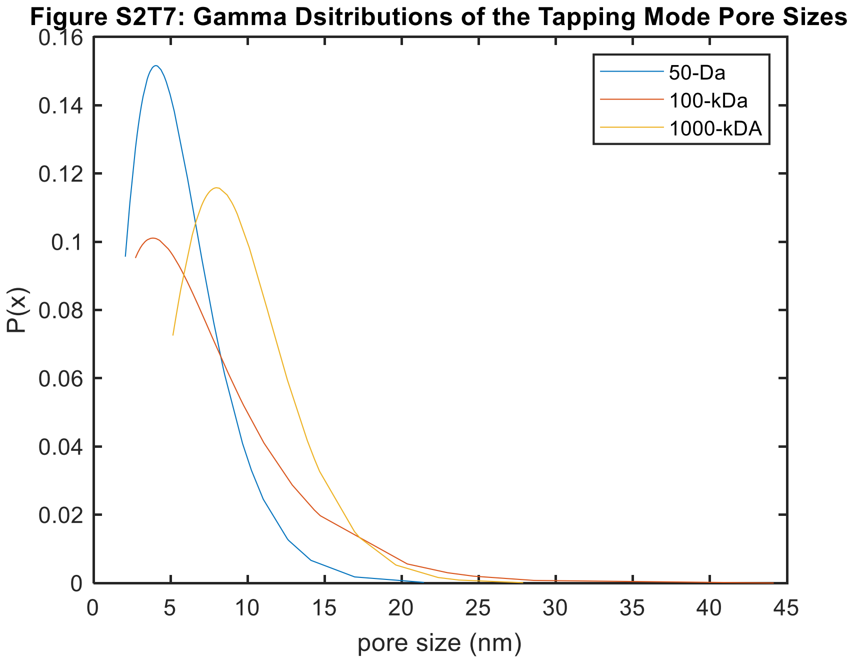

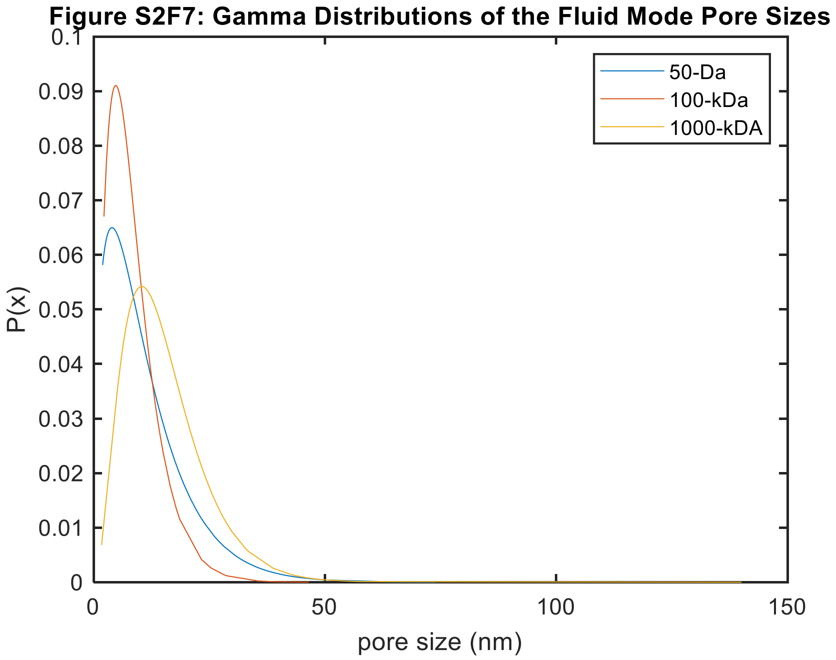

Figure S2. (C) Raw datasets fitted Gamma Distributions (Figure S2T7 tapping mode; Figure S2F7 fluid mode).

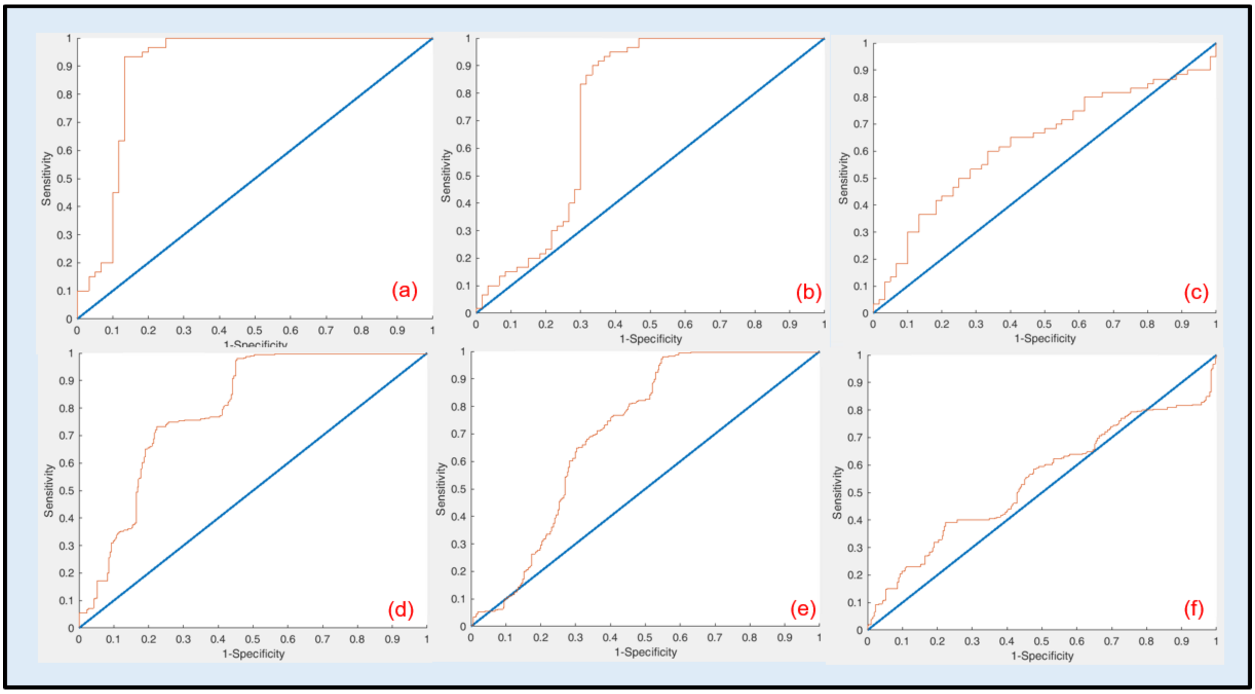

Figure S3. Receiver operating curves applied to the raw datasets . Top and bottom rows correspond to tapping and fluid modes, respectively: (**a**, **d**) 100 kDa vs 1000 kDa in tapping and fluid modes; (**b**, **e**) 50 kDa vs 1000 kDa in tapping and fluid modes; (**c, f**) 50 kDa vs 100 kDa in tapping and fluid modes. Corresponding AUCs are presented in Table 7.
